# 7-Iodo-3,3-diphenyl­octa­hydro­benzo­furan

**DOI:** 10.1107/S1600536813007563

**Published:** 2013-03-23

**Authors:** Muhammad Sohail, Wang Yao-Feng, Wang Qi, Fu-Xue Chen

**Affiliations:** a School of Chemical Engineering and Environment, Beijing Institue of Technology, Beijing 100081, People’s Republic of China

## Abstract

The title compound, C_20_H_21_IO, was synthesized by cyclo­haloetherification of 2-(cyclo­hex-2-en­yl)-2,2-diphenyl­ethanol in CH_2_Cl_2_, and crystallized with two independent mol­ecules in the asymmetric unit. The six-membered cyclo­hexane ring adopts a chair conformation, while the five-membered ring adopts an envelope conformation with the fused C atom opposite the O atom as the flap in each case [displacements of the flap atoms = 0.6813 (3) and 0.6679 (3) Å]. In the crystal, mol­ecules are linked *via* pairs of C—H⋯π inter­actions, forming inversion dimers.

## Related literature

For the title compound as a core structure of many drugs and natural products, see: Huang & Chen (2007 ▶); Trost *et al.* (2003 ▶). For the synthesis of 2-(cyclo­hex-2-en­yl)-2,2-diphenyl­ethanol, see: Brooner & Widenhoefer (2011 ▶).
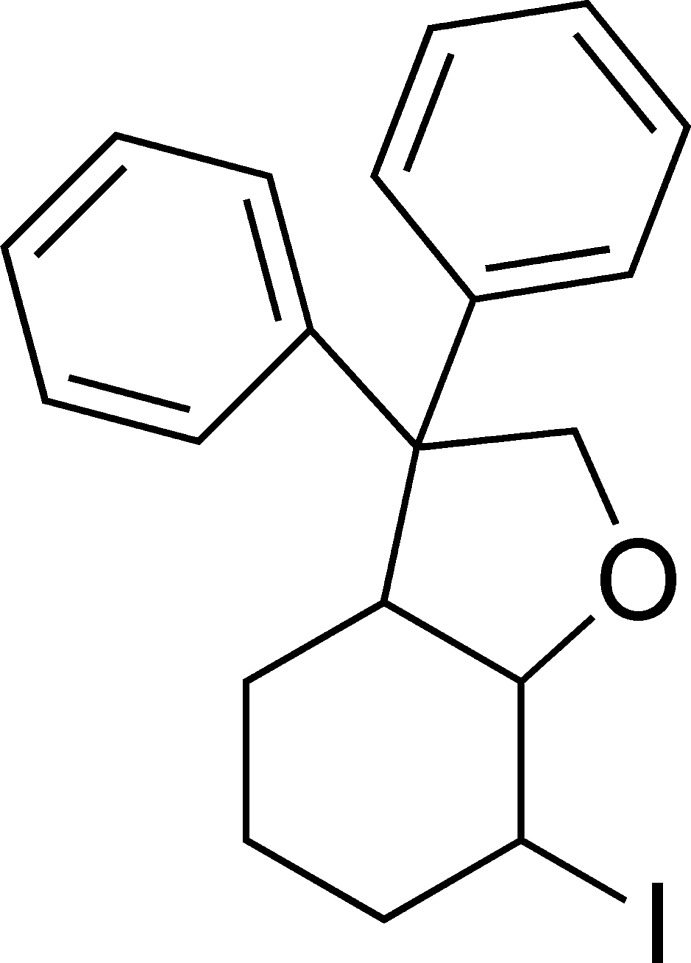


## Experimental

### 

#### Crystal data

C_20_H_21_IO
*M* *_r_* = 404.27
Triclinic, 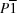

*a* = 11.4082 (18) Å
*b* = 12.523 (2) Å
*c* = 14.007 (3) Å
α = 73.306 (8)°
β = 71.646 (8)°
γ = 64.945 (7)°
*V* = 1692.8 (5) Å^3^
*Z* = 4
Mo *K*α radiation
μ = 1.89 mm^−1^
*T* = 153 K
0.33 × 0.27 × 0.10 mm

#### Data collection

Rigaku AFC10/Saturn724+ diffractometer
Absorption correction: multi-scan (*CrystalClear*; Rigaku, 2008 ▶) *T*_min_ = 0.572, *T*_max_ = 0.833
19455 measured reflections
9610 independent reflections
7599 reflections with *I* > 2σ(*I*)
*R*_int_ = 0.034

#### Refinement

*R*[*F* ^2^ > 2σ(*F* ^2^)] = 0.039
*wR*(*F* ^2^) = 0.091
*S* = 1.00
9610 reflections
397 parameters
H-atom parameters constrained
Δρ_max_ = 1.05 e Å^−3^
Δρ_min_ = −0.75 e Å^−3^

### 

Data collection: *CrystalClear* (Rigaku, 2008 ▶); cell refinement: *CrystalClear*; data reduction: *CrystalClear*; program(s) used to solve structure: *SHELXS97* (Sheldrick, 2008 ▶); program(s) used to refine structure: *SHELXL97* (Sheldrick, 2008 ▶); molecular graphics: *SHELXTL* (Sheldrick, 2008 ▶); software used to prepare material for publication: *SHELXTL*.

## Supplementary Material

Crystal structure: contains datablock(s) I, global. DOI: 10.1107/S1600536813007563/hg5298sup1.cif

Structure factors: contains datablock(s) I. DOI: 10.1107/S1600536813007563/hg5298Isup2.hkl

Supplementary material file. DOI: 10.1107/S1600536813007563/hg5298Isup3.cml

Additional supplementary materials:  crystallographic information; 3D view; checkCIF report

## Figures and Tables

**Table 1:** Hydrogen-bond geometry (Å, °) *Cg*1 and *Cg*2 are the centroids of the C9–C14 and C15′–C20′ rings, respectively.

*D*—H⋯*A*	*D*—H	H⋯*A*	*D*⋯*A*	*D*—H⋯*A*
C2—H2⋯*Cg*1^i^	1.00	2.53	3.519 (3)	171
C2′—H2′⋯*Cg*2^ii^	1.00	2.54	3.533 (3)	171

Symmetry codes: (i) 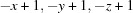; (ii) 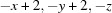.

**Fig. 1.**
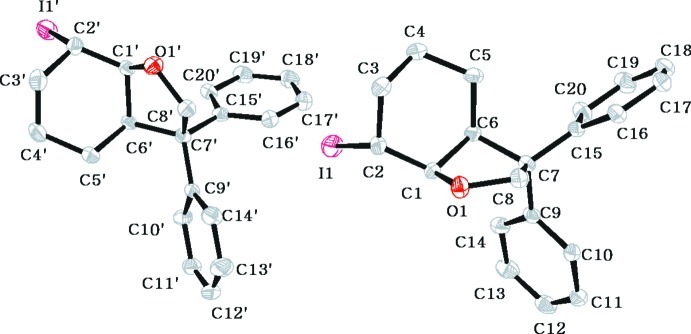
The molecular structure of the two molecules of (I), with atom labels and 30% probability displacement ellipsoids for non-H atoms.

**Fig. 2.**
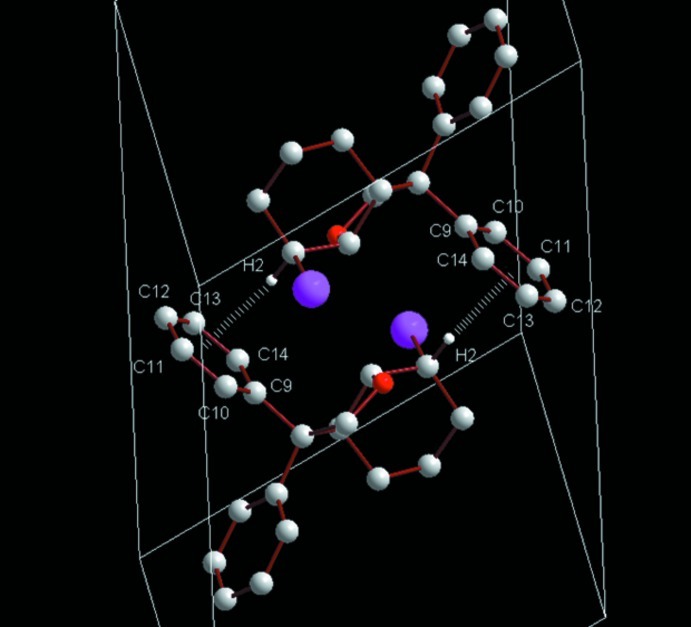
A view of C—H···π interactions are indicated by dotted lines in the crystal structure of the title compound.

## supplementary crystallographic information

### Comment

The title compound (I, Fig. 1), is an important core structure of many organic drugs and natural products (Huang *et al.* 2007, Trost *et al.*, 2003) and is useful to introduce functionality at C7. The asymmetric unit of title compound consists of two independent molecules in which the iodo-cyclohexane rings adopt chair conformations. In the crystal lattice, two molecules in asymmetric unit are linked by C—H···π interactions with phenyl ring.

### Experimental

N-Iodosuccinimide (13.5 mg, 0.06 mmole, 1.2 eq) was added to the solution of 2-(cyclohex-2-enyl)-2,2-diphenylethanol (13.9 mg, 0.05 mmole, 1 eq) in CH_2_Cl_2_ (0.5 ml) at -78°C. The reaction mixture was stirred at -78°C for 2.5 h, after reaction completion, as monitored by TLC the crude was directly loaded on column and purified by flash column chromatography (silica gel, Et_2_O-Petrolium ether, 1:40), redissolving of crude in *n*-hexane afforded pure crystals (99%) of (I) (Brooner *et al.* 2011).

### Refinement

Carbon protons were included in the riding model approximation with C—H distances 0.95-1.00 Å, and with U_iso_(H)=1.2U_eq_(C).

### Crystal data

**Table:**

C_20_H_21_IO	*Z* = 4
*M**_r_* = 404.27	*F*(000) = 808
Triclinic, *P*1	*D*_x_ = 1.586 Mg m^−^^3^
Hall symbol: -P 1	Mo *K*α radiation, λ = 0.71073 Å
*a* = 11.4082 (18) Å	Cell parameters from 5864 reflections
*b* = 12.523 (2) Å	θ = 2.2–30.0°
*c* = 14.007 (3) Å	µ = 1.89 mm^−^^1^
α = 73.306 (8)°	*T* = 153 K
β = 71.646 (8)°	Block, colourless
γ = 64.945 (7)°	0.33 × 0.27 × 0.10 mm
*V* = 1692.8 (5) Å^3^	

### Data collection

**Table:**

Rigaku AFC10/Saturn724+ diffractometer	9610 independent reflections
Radiation source: Rotating Anode	7599 reflections with *I* > 2σ(*I*)
Graphite monochromator	*R*_int_ = 0.034
Detector resolution: 28.5714 pixels mm^-1^	θ_max_ = 30.0°, θ_min_ = 2.2°
phi and ω scans	*h* = −16→13
Absorption correction: multi-scan (*CrystalClear*; Rigaku, 2008)	*k* = −17→16
*T*_min_ = 0.572, *T*_max_ = 0.833	*l* = −19→19
19455 measured reflections	

### Refinement

**Table:**

Refinement on *F*^2^	Primary atom site location: structure-invariant direct methods
Least-squares matrix: full	Secondary atom site location: difference Fourier map
*R*[*F*^2^ > 2σ(*F*^2^)] = 0.039	Hydrogen site location: inferred from neighbouring sites
*wR*(*F*^2^) = 0.091	H-atom parameters constrained
*S* = 1.00	*w* = 1/[σ^2^(*F*_o_^2^) + (0.0318*P*)^2^ + 1.060*P*] where *P* = (*F*_o_^2^ + 2*F*_c_^2^)/3
9610 reflections	(Δ/σ)_max_ = 0.002
397 parameters	Δρ_max_ = 1.05 e Å^−^^3^
0 restraints	Δρ_min_ = −0.75 e Å^−^^3^

### Special details

**Table:**

Geometry. All e.s.d.'s (except the e.s.d. in the dihedral angle between two l.s. planes) are estimated using the full covariance matrix. The cell e.s.d.'s are taken into account individually in the estimation of e.s.d.'s in distances, angles and torsion angles; correlations between e.s.d.'s in cell parameters are only used when they are defined by crystal symmetry. An approximate (isotropic) treatment of cell e.s.d.'s is used for estimating e.s.d.'s involving l.s. planes.
Refinement. Refinement of *F*^2^ against ALL reflections. The weighted *R*-factor *wR* and goodness of fit *S* are based on *F*^2^, conventional *R*-factors *R* are based on *F*, with *F* set to zero for negative *F*^2^. The threshold expression of *F*^2^ > σ(*F*^2^) is used only for calculating *R*-factors(gt) *etc*. and is not relevant to the choice of reflections for refinement. *R*-factors based on *F*^2^ are statistically about twice as large as those based on *F*, and *R*- factors based on ALL data will be even larger.

### Fractional atomic coordinates and isotropic or equivalent isotropic displacement parameters (Å^2^)

**Table:**

	*x*	*y*	*z*	*U*_iso_*/*U*_eq_	
I1	0.65947 (2)	0.703016 (18)	0.295052 (16)	0.03449 (6)	
O1	0.53230 (19)	0.40425 (16)	0.40367 (15)	0.0228 (4)	
C1	0.5264 (2)	0.5248 (2)	0.3578 (2)	0.0184 (5)	
H1	0.4776	0.5777	0.4103	0.022*	
C2	0.6681 (3)	0.5206 (2)	0.3193 (2)	0.0216 (5)	
H2	0.7177	0.4710	0.3743	0.026*	
C3	0.7423 (3)	0.4681 (3)	0.2228 (2)	0.0278 (6)	
H3A	0.7611	0.3809	0.2388	0.033*	
H3B	0.8283	0.4796	0.1973	0.033*	
C4	0.6631 (3)	0.5265 (3)	0.1397 (2)	0.0285 (6)	
H4A	0.7133	0.4889	0.0784	0.034*	
H4B	0.6493	0.6127	0.1201	0.034*	
C5	0.5288 (3)	0.5116 (3)	0.1782 (2)	0.0228 (5)	
H5A	0.5438	0.4253	0.1913	0.027*	
H5B	0.4778	0.5523	0.1240	0.027*	
C6	0.4460 (2)	0.5624 (2)	0.27622 (19)	0.0176 (5)	
H6	0.4037	0.6517	0.2601	0.021*	
C7	0.3390 (2)	0.5062 (2)	0.33450 (19)	0.0176 (5)	
C8	0.4302 (3)	0.3834 (2)	0.3831 (2)	0.0212 (5)	
H8A	0.3792	0.3500	0.4472	0.025*	
H8B	0.4686	0.3260	0.3355	0.025*	
C9	0.2348 (2)	0.5739 (2)	0.4198 (2)	0.0196 (5)	
C10	0.1516 (3)	0.5193 (3)	0.4926 (2)	0.0241 (6)	
H10	0.1619	0.4411	0.4891	0.029*	
C11	0.0547 (3)	0.5757 (3)	0.5698 (2)	0.0304 (7)	
H11	0.0001	0.5360	0.6187	0.036*	
C12	0.0376 (3)	0.6890 (3)	0.5754 (2)	0.0314 (7)	
H12	−0.0294	0.7283	0.6279	0.038*	
C13	0.1182 (3)	0.7460 (3)	0.5043 (2)	0.0302 (7)	
H13	0.1067	0.8244	0.5082	0.036*	
C14	0.2156 (3)	0.6888 (3)	0.4276 (2)	0.0241 (6)	
H14	0.2704	0.7288	0.3794	0.029*	
C15	0.2651 (2)	0.4991 (2)	0.2641 (2)	0.0195 (5)	
C16	0.2684 (3)	0.3913 (3)	0.2510 (2)	0.0250 (6)	
H16	0.3194	0.3175	0.2864	0.030*	
C17	0.1972 (3)	0.3913 (3)	0.1862 (2)	0.0300 (7)	
H17	0.2004	0.3174	0.1777	0.036*	
C18	0.1223 (3)	0.4975 (3)	0.1342 (2)	0.0296 (6)	
H18	0.0736	0.4969	0.0905	0.036*	
C19	0.1185 (3)	0.6049 (3)	0.1461 (2)	0.0311 (7)	
H19	0.0671	0.6783	0.1104	0.037*	
C20	0.1893 (3)	0.6057 (3)	0.2100 (2)	0.0260 (6)	
H20	0.1863	0.6800	0.2172	0.031*	
I1'	1.19069 (2)	1.181469 (19)	0.013330 (16)	0.03506 (7)	
O1'	1.02918 (19)	0.90326 (17)	0.12779 (15)	0.0251 (4)	
C1'	1.0347 (3)	1.0201 (2)	0.1134 (2)	0.0203 (5)	
H1'	0.9883	1.0756	0.0581	0.024*	
C2'	1.1807 (3)	1.0044 (3)	0.0810 (2)	0.0245 (6)	
H2'	1.2252	0.9533	0.0270	0.029*	
C3'	1.2537 (3)	0.9465 (3)	0.1671 (2)	0.0305 (7)	
H3'1	1.3435	0.9502	0.1426	0.037*	
H3'2	1.2639	0.8610	0.1875	0.037*	
C4'	1.1806 (3)	1.0078 (3)	0.2598 (2)	0.0301 (6)	
H4'1	1.1757	1.0918	0.2411	0.036*	
H4'2	1.2300	0.9663	0.3150	0.036*	
C5'	1.0407 (3)	1.0052 (3)	0.2979 (2)	0.0256 (6)	
H5'1	0.9939	1.0483	0.3563	0.031*	
H5'2	1.0471	0.9210	0.3230	0.031*	
C6'	0.9582 (3)	1.0620 (2)	0.21581 (19)	0.0187 (5)	
H6'	0.9238	1.1514	0.2063	0.022*	
C7'	0.8417 (3)	1.0166 (2)	0.2427 (2)	0.0187 (5)	
C8'	0.9195 (3)	0.8919 (2)	0.2105 (2)	0.0229 (6)	
H8'1	0.9521	0.8306	0.2686	0.027*	
H8'2	0.8617	0.8680	0.1878	0.027*	
C9'	0.7693 (3)	1.0112 (2)	0.3550 (2)	0.0195 (5)	
C10'	0.7127 (3)	1.1150 (2)	0.3963 (2)	0.0238 (6)	
H10'	0.7222	1.1868	0.3546	0.029*	
C11'	0.6425 (3)	1.1161 (3)	0.4972 (2)	0.0273 (6)	
H11'	0.6047	1.1879	0.5242	0.033*	
C12'	0.6277 (3)	1.0117 (3)	0.5583 (2)	0.0322 (7)	
H12'	0.5803	1.0116	0.6276	0.039*	
C13'	0.6820 (3)	0.9087 (3)	0.5183 (2)	0.0368 (7)	
H13'	0.6707	0.8376	0.5600	0.044*	
C14'	0.7535 (3)	0.9072 (3)	0.4174 (2)	0.0295 (6)	
H14'	0.7916	0.8349	0.3911	0.035*	
C15'	0.7364 (3)	1.0920 (2)	0.1781 (2)	0.0198 (5)	
C16'	0.6360 (3)	1.0507 (3)	0.1877 (2)	0.0271 (6)	
H16'	0.6351	0.9781	0.2331	0.032*	
C17'	0.5377 (3)	1.1134 (3)	0.1324 (2)	0.0333 (7)	
H17'	0.4708	1.0836	0.1398	0.040*	
C18'	0.5376 (3)	1.2199 (3)	0.0662 (2)	0.0332 (7)	
H18'	0.4704	1.2634	0.0283	0.040*	
C19'	0.6352 (3)	1.2623 (3)	0.0557 (2)	0.0288 (6)	
H19'	0.6356	1.3348	0.0100	0.035*	
C20'	0.7336 (3)	1.1995 (2)	0.1118 (2)	0.0232 (6)	
H20'	0.7995	1.2304	0.1047	0.028*	

### Atomic displacement parameters (Å^2^)

**Table:**

	*U*^11^	*U*^22^	*U*^33^	*U*^12^	*U*^13^	*U*^23^
I1	0.04589 (13)	0.03359 (11)	0.03171 (11)	−0.02655 (10)	−0.00455 (9)	−0.00285 (9)
O1	0.0265 (10)	0.0197 (9)	0.0248 (10)	−0.0122 (8)	−0.0108 (8)	0.0038 (8)
C1	0.0200 (12)	0.0202 (12)	0.0166 (12)	−0.0102 (10)	−0.0033 (9)	−0.0018 (10)
C2	0.0205 (12)	0.0250 (13)	0.0213 (13)	−0.0099 (11)	−0.0060 (10)	−0.0033 (11)
C3	0.0201 (13)	0.0360 (16)	0.0271 (15)	−0.0110 (12)	−0.0010 (11)	−0.0089 (13)
C4	0.0257 (14)	0.0394 (17)	0.0210 (14)	−0.0141 (13)	0.0013 (11)	−0.0102 (12)
C5	0.0228 (13)	0.0294 (14)	0.0161 (12)	−0.0088 (11)	−0.0029 (10)	−0.0063 (11)
C6	0.0182 (12)	0.0199 (12)	0.0147 (11)	−0.0071 (10)	−0.0038 (9)	−0.0026 (9)
C7	0.0196 (12)	0.0174 (12)	0.0169 (12)	−0.0059 (10)	−0.0055 (9)	−0.0046 (9)
C8	0.0210 (12)	0.0212 (13)	0.0228 (13)	−0.0086 (10)	−0.0065 (10)	−0.0029 (10)
C9	0.0153 (11)	0.0247 (13)	0.0187 (12)	−0.0040 (10)	−0.0063 (9)	−0.0060 (10)
C10	0.0191 (12)	0.0350 (15)	0.0222 (13)	−0.0129 (11)	−0.0027 (10)	−0.0083 (12)
C11	0.0211 (13)	0.054 (2)	0.0215 (14)	−0.0184 (14)	0.0009 (11)	−0.0130 (14)
C12	0.0198 (13)	0.0466 (18)	0.0260 (15)	−0.0042 (13)	−0.0041 (11)	−0.0176 (14)
C13	0.0314 (15)	0.0264 (15)	0.0288 (15)	−0.0011 (12)	−0.0084 (12)	−0.0124 (12)
C14	0.0249 (13)	0.0256 (14)	0.0211 (13)	−0.0072 (11)	−0.0046 (10)	−0.0068 (11)
C15	0.0177 (12)	0.0240 (13)	0.0181 (12)	−0.0081 (10)	−0.0014 (9)	−0.0080 (10)
C16	0.0250 (13)	0.0317 (15)	0.0224 (13)	−0.0118 (12)	−0.0052 (11)	−0.0089 (12)
C17	0.0334 (16)	0.0384 (17)	0.0280 (15)	−0.0209 (14)	−0.0007 (12)	−0.0146 (13)
C18	0.0249 (14)	0.0471 (18)	0.0237 (14)	−0.0149 (13)	−0.0040 (11)	−0.0153 (13)
C19	0.0263 (14)	0.0367 (17)	0.0303 (16)	−0.0055 (13)	−0.0131 (12)	−0.0080 (13)
C20	0.0242 (14)	0.0264 (14)	0.0295 (15)	−0.0054 (11)	−0.0106 (11)	−0.0089 (12)
I1'	0.04789 (13)	0.04025 (12)	0.02762 (11)	−0.02840 (10)	−0.00919 (9)	−0.00140 (9)
O1'	0.0276 (10)	0.0228 (10)	0.0267 (10)	−0.0111 (8)	0.0004 (8)	−0.0114 (8)
C1'	0.0239 (13)	0.0226 (13)	0.0178 (12)	−0.0106 (11)	−0.0045 (10)	−0.0054 (10)
C2'	0.0231 (13)	0.0277 (14)	0.0253 (14)	−0.0119 (11)	−0.0034 (11)	−0.0070 (11)
C3'	0.0236 (14)	0.0333 (16)	0.0356 (17)	−0.0095 (12)	−0.0092 (12)	−0.0064 (13)
C4'	0.0323 (15)	0.0389 (17)	0.0255 (15)	−0.0156 (13)	−0.0140 (12)	−0.0026 (13)
C5'	0.0287 (14)	0.0316 (15)	0.0187 (13)	−0.0120 (12)	−0.0084 (11)	−0.0028 (11)
C6'	0.0228 (12)	0.0205 (12)	0.0164 (12)	−0.0098 (10)	−0.0043 (10)	−0.0054 (10)
C7'	0.0220 (12)	0.0170 (12)	0.0178 (12)	−0.0071 (10)	−0.0050 (10)	−0.0038 (10)
C8'	0.0238 (13)	0.0207 (13)	0.0243 (13)	−0.0077 (11)	−0.0044 (11)	−0.0059 (11)
C9'	0.0204 (12)	0.0217 (13)	0.0168 (12)	−0.0080 (10)	−0.0079 (10)	0.0003 (10)
C10'	0.0272 (14)	0.0217 (13)	0.0193 (13)	−0.0082 (11)	−0.0047 (10)	−0.0008 (10)
C11'	0.0246 (14)	0.0322 (15)	0.0232 (14)	−0.0079 (12)	−0.0044 (11)	−0.0069 (12)
C12'	0.0301 (15)	0.0488 (19)	0.0180 (13)	−0.0184 (14)	−0.0041 (11)	−0.0018 (13)
C13'	0.0483 (19)	0.0392 (18)	0.0256 (16)	−0.0266 (16)	−0.0056 (14)	0.0037 (13)
C14'	0.0421 (17)	0.0270 (15)	0.0247 (15)	−0.0193 (13)	−0.0089 (13)	−0.0004 (12)
C15'	0.0212 (12)	0.0192 (12)	0.0183 (12)	−0.0051 (10)	−0.0035 (10)	−0.0069 (10)
C16'	0.0264 (14)	0.0330 (15)	0.0233 (14)	−0.0133 (12)	−0.0041 (11)	−0.0048 (12)
C17'	0.0239 (14)	0.0474 (19)	0.0316 (16)	−0.0135 (14)	−0.0066 (12)	−0.0103 (14)
C18'	0.0244 (14)	0.0414 (18)	0.0288 (16)	−0.0018 (13)	−0.0119 (12)	−0.0084 (14)
C19'	0.0318 (15)	0.0249 (14)	0.0243 (14)	−0.0032 (12)	−0.0114 (12)	−0.0023 (11)
C20'	0.0259 (13)	0.0242 (13)	0.0199 (13)	−0.0081 (11)	−0.0073 (10)	−0.0038 (11)

### Geometric parameters (Å, º)

**Table:**

I1—C2	2.175 (3)	I1'—C2'	2.183 (3)
O1—C8	1.421 (3)	O1'—C8'	1.439 (3)
O1—C1	1.442 (3)	O1'—C1'	1.444 (3)
C1—C2	1.518 (4)	C1'—C2'	1.522 (4)
C1—C6	1.534 (4)	C1'—C6'	1.534 (3)
C1—H1	1.0000	C1'—H1'	1.0000
C2—C3	1.514 (4)	C2'—C3'	1.515 (4)
C2—H2	1.0000	C2'—H2'	1.0000
C3—C4	1.524 (4)	C3'—C4'	1.523 (4)
C3—H3A	0.9900	C3'—H3'1	0.9900
C3—H3B	0.9900	C3'—H3'2	0.9900
C4—C5	1.528 (4)	C4'—C5'	1.526 (4)
C4—H4A	0.9900	C4'—H4'1	0.9900
C4—H4B	0.9900	C4'—H4'2	0.9900
C5—C6	1.536 (3)	C5'—C6'	1.535 (4)
C5—H5A	0.9900	C5'—H5'1	0.9900
C5—H5B	0.9900	C5'—H5'2	0.9900
C6—C7	1.556 (3)	C6'—C7'	1.560 (4)
C6—H6	1.0000	C6'—H6'	1.0000
C7—C15	1.526 (4)	C7'—C9'	1.527 (4)
C7—C9	1.547 (3)	C7'—C15'	1.545 (4)
C7—C8	1.550 (4)	C7'—C8'	1.553 (4)
C8—H8A	0.9900	C8'—H8'1	0.9900
C8—H8B	0.9900	C8'—H8'2	0.9900
C9—C14	1.393 (4)	C9'—C10'	1.388 (4)
C9—C10	1.396 (4)	C9'—C14'	1.392 (4)
C10—C11	1.384 (4)	C10'—C11'	1.389 (4)
C10—H10	0.9500	C10'—H10'	0.9500
C11—C12	1.370 (5)	C11'—C12'	1.386 (4)
C11—H11	0.9500	C11'—H11'	0.9500
C12—C13	1.385 (4)	C12'—C13'	1.372 (5)
C12—H12	0.9500	C12'—H12'	0.9500
C13—C14	1.385 (4)	C13'—C14'	1.392 (4)
C13—H13	0.9500	C13'—H13'	0.9500
C14—H14	0.9500	C14'—H14'	0.9500
C15—C16	1.398 (4)	C15'—C20'	1.393 (4)
C15—C20	1.399 (4)	C15'—C16'	1.401 (4)
C16—C17	1.395 (4)	C16'—C17'	1.388 (4)
C16—H16	0.9500	C16'—H16'	0.9500
C17—C18	1.380 (5)	C17'—C18'	1.388 (5)
C17—H17	0.9500	C17'—H17'	0.9500
C18—C19	1.383 (4)	C18'—C19'	1.378 (4)
C18—H18	0.9500	C18'—H18'	0.9500
C19—C20	1.386 (4)	C19'—C20'	1.396 (4)
C19—H19	0.9500	C19'—H19'	0.9500
C20—H20	0.9500	C20'—H20'	0.9500
			
C8—O1—C1	110.04 (19)	C8'—O1'—C1'	109.76 (19)
O1—C1—C2	107.4 (2)	O1'—C1'—C2'	107.0 (2)
O1—C1—C6	104.0 (2)	O1'—C1'—C6'	104.5 (2)
C2—C1—C6	116.3 (2)	C2'—C1'—C6'	116.3 (2)
O1—C1—H1	109.6	O1'—C1'—H1'	109.6
C2—C1—H1	109.6	C2'—C1'—H1'	109.6
C6—C1—H1	109.6	C6'—C1'—H1'	109.6
C3—C2—C1	114.5 (2)	C3'—C2'—C1'	114.1 (2)
C3—C2—I1	110.48 (19)	C3'—C2'—I1'	110.34 (19)
C1—C2—I1	107.01 (17)	C1'—C2'—I1'	107.33 (18)
C3—C2—H2	108.2	C3'—C2'—H2'	108.3
C1—C2—H2	108.2	C1'—C2'—H2'	108.3
I1—C2—H2	108.2	I1'—C2'—H2'	108.3
C2—C3—C4	111.7 (2)	C2'—C3'—C4'	112.0 (2)
C2—C3—H3A	109.3	C2'—C3'—H3'1	109.2
C4—C3—H3A	109.3	C4'—C3'—H3'1	109.2
C2—C3—H3B	109.3	C2'—C3'—H3'2	109.2
C4—C3—H3B	109.3	C4'—C3'—H3'2	109.2
H3A—C3—H3B	108.0	H3'1—C3'—H3'2	107.9
C3—C4—C5	110.1 (2)	C3'—C4'—C5'	110.2 (2)
C3—C4—H4A	109.6	C3'—C4'—H4'1	109.6
C5—C4—H4A	109.6	C5'—C4'—H4'1	109.6
C3—C4—H4B	109.6	C3'—C4'—H4'2	109.6
C5—C4—H4B	109.6	C5'—C4'—H4'2	109.6
H4A—C4—H4B	108.2	H4'1—C4'—H4'2	108.1
C4—C5—C6	113.5 (2)	C4'—C5'—C6'	113.6 (2)
C4—C5—H5A	108.9	C4'—C5'—H5'1	108.8
C6—C5—H5A	108.9	C6'—C5'—H5'1	108.8
C4—C5—H5B	108.9	C4'—C5'—H5'2	108.8
C6—C5—H5B	108.9	C6'—C5'—H5'2	108.8
H5A—C5—H5B	107.7	H5'1—C5'—H5'2	107.7
C1—C6—C5	112.9 (2)	C1'—C6'—C5'	112.9 (2)
C1—C6—C7	100.37 (19)	C1'—C6'—C7'	100.5 (2)
C5—C6—C7	111.6 (2)	C5'—C6'—C7'	111.4 (2)
C1—C6—H6	110.5	C1'—C6'—H6'	110.6
C5—C6—H6	110.5	C5'—C6'—H6'	110.6
C7—C6—H6	110.5	C7'—C6'—H6'	110.6
C15—C7—C9	108.0 (2)	C9'—C7'—C15'	107.4 (2)
C15—C7—C8	114.7 (2)	C9'—C7'—C8'	113.8 (2)
C9—C7—C8	109.6 (2)	C15'—C7'—C8'	109.0 (2)
C15—C7—C6	112.9 (2)	C9'—C7'—C6'	113.4 (2)
C9—C7—C6	113.0 (2)	C15'—C7'—C6'	113.6 (2)
C8—C7—C6	98.61 (19)	C8'—C7'—C6'	99.6 (2)
O1—C8—C7	106.6 (2)	O1'—C8'—C7'	106.6 (2)
O1—C8—H8A	110.4	O1'—C8'—H8'1	110.4
C7—C8—H8A	110.4	C7'—C8'—H8'1	110.4
O1—C8—H8B	110.4	O1'—C8'—H8'2	110.4
C7—C8—H8B	110.4	C7'—C8'—H8'2	110.4
H8A—C8—H8B	108.6	H8'1—C8'—H8'2	108.6
C14—C9—C10	117.0 (2)	C10'—C9'—C14'	118.3 (3)
C14—C9—C7	124.0 (2)	C10'—C9'—C7'	118.9 (2)
C10—C9—C7	118.9 (2)	C14'—C9'—C7'	122.8 (2)
C11—C10—C9	121.9 (3)	C9'—C10'—C11'	121.4 (3)
C11—C10—H10	119.0	C9'—C10'—H10'	119.3
C9—C10—H10	119.0	C11'—C10'—H10'	119.3
C12—C11—C10	119.9 (3)	C12'—C11'—C10'	119.6 (3)
C12—C11—H11	120.1	C12'—C11'—H11'	120.2
C10—C11—H11	120.1	C10'—C11'—H11'	120.2
C11—C12—C13	119.8 (3)	C13'—C12'—C11'	119.6 (3)
C11—C12—H12	120.1	C13'—C12'—H12'	120.2
C13—C12—H12	120.1	C11'—C12'—H12'	120.2
C14—C13—C12	120.2 (3)	C12'—C13'—C14'	120.8 (3)
C14—C13—H13	119.9	C12'—C13'—H13'	119.6
C12—C13—H13	119.9	C14'—C13'—H13'	119.6
C13—C14—C9	121.3 (3)	C9'—C14'—C13'	120.2 (3)
C13—C14—H14	119.4	C9'—C14'—H14'	119.9
C9—C14—H14	119.4	C13'—C14'—H14'	119.9
C16—C15—C20	117.9 (3)	C20'—C15'—C16'	117.7 (3)
C16—C15—C7	123.5 (2)	C20'—C15'—C7'	124.2 (2)
C20—C15—C7	118.7 (2)	C16'—C15'—C7'	118.1 (2)
C17—C16—C15	120.5 (3)	C17'—C16'—C15'	121.5 (3)
C17—C16—H16	119.7	C17'—C16'—H16'	119.2
C15—C16—H16	119.7	C15'—C16'—H16'	119.2
C18—C17—C16	120.6 (3)	C18'—C17'—C16'	119.7 (3)
C18—C17—H17	119.7	C18'—C17'—H17'	120.1
C16—C17—H17	119.7	C16'—C17'—H17'	120.1
C17—C18—C19	119.5 (3)	C19'—C18'—C17'	119.7 (3)
C17—C18—H18	120.2	C19'—C18'—H18'	120.1
C19—C18—H18	120.2	C17'—C18'—H18'	120.1
C18—C19—C20	120.2 (3)	C18'—C19'—C20'	120.5 (3)
C18—C19—H19	119.9	C18'—C19'—H19'	119.8
C20—C19—H19	119.9	C20'—C19'—H19'	119.8
C19—C20—C15	121.3 (3)	C15'—C20'—C19'	120.8 (3)
C19—C20—H20	119.4	C15'—C20'—H20'	119.6
C15—C20—H20	119.4	C19'—C20'—H20'	119.6
			
C8—O1—C1—C2	−143.3 (2)	C8'—O1'—C1'—C2'	145.4 (2)
C8—O1—C1—C6	−19.5 (3)	C8'—O1'—C1'—C6'	21.4 (3)
O1—C1—C2—C3	74.7 (3)	O1'—C1'—C2'—C3'	−74.9 (3)
C6—C1—C2—C3	−41.2 (3)	C6'—C1'—C2'—C3'	41.4 (3)
O1—C1—C2—I1	−162.47 (16)	O1'—C1'—C2'—I1'	162.51 (16)
C6—C1—C2—I1	81.6 (2)	C6'—C1'—C2'—I1'	−81.2 (2)
C1—C2—C3—C4	50.6 (3)	C1'—C2'—C3'—C4'	−50.7 (3)
I1—C2—C3—C4	−70.3 (3)	I1'—C2'—C3'—C4'	70.2 (3)
C2—C3—C4—C5	−58.0 (3)	C2'—C3'—C4'—C5'	57.8 (3)
C3—C4—C5—C6	56.8 (3)	C3'—C4'—C5'—C6'	−56.4 (3)
O1—C1—C6—C5	−79.3 (2)	O1'—C1'—C6'—C5'	79.0 (3)
C2—C1—C6—C5	38.5 (3)	C2'—C1'—C6'—C5'	−38.7 (3)
O1—C1—C6—C7	39.7 (2)	O1'—C1'—C6'—C7'	−39.8 (2)
C2—C1—C6—C7	157.5 (2)	C2'—C1'—C6'—C7'	−157.4 (2)
C4—C5—C6—C1	−46.6 (3)	C4'—C5'—C6'—C1'	46.5 (3)
C4—C5—C6—C7	−158.8 (2)	C4'—C5'—C6'—C7'	158.6 (2)
C1—C6—C7—C15	−164.5 (2)	C1'—C6'—C7'—C9'	162.8 (2)
C5—C6—C7—C15	−44.6 (3)	C5'—C6'—C7'—C9'	43.0 (3)
C1—C6—C7—C9	72.6 (2)	C1'—C6'—C7'—C15'	−74.1 (2)
C5—C6—C7—C9	−167.5 (2)	C5'—C6'—C7'—C15'	166.0 (2)
C1—C6—C7—C8	−43.0 (2)	C1'—C6'—C7'—C8'	41.6 (2)
C5—C6—C7—C8	76.9 (2)	C5'—C6'—C7'—C8'	−78.2 (2)
C1—O1—C8—C7	−9.2 (3)	C1'—O1'—C8'—C7'	6.3 (3)
C15—C7—C8—O1	153.3 (2)	C9'—C7'—C8'—O1'	−151.4 (2)
C9—C7—C8—O1	−85.2 (2)	C15'—C7'—C8'—O1'	88.8 (3)
C6—C7—C8—O1	33.1 (2)	C6'—C7'—C8'—O1'	−30.4 (3)
C15—C7—C9—C14	−109.6 (3)	C15'—C7'—C9'—C10'	−68.6 (3)
C8—C7—C9—C14	124.8 (3)	C8'—C7'—C9'—C10'	170.7 (2)
C6—C7—C9—C14	16.0 (4)	C6'—C7'—C9'—C10'	57.8 (3)
C15—C7—C9—C10	68.7 (3)	C15'—C7'—C9'—C14'	109.2 (3)
C8—C7—C9—C10	−56.9 (3)	C8'—C7'—C9'—C14'	−11.6 (4)
C6—C7—C9—C10	−165.8 (2)	C6'—C7'—C9'—C14'	−124.4 (3)
C14—C9—C10—C11	−0.4 (4)	C14'—C9'—C10'—C11'	0.3 (4)
C7—C9—C10—C11	−178.8 (2)	C7'—C9'—C10'—C11'	178.2 (3)
C9—C10—C11—C12	0.6 (4)	C9'—C10'—C11'—C12'	−0.3 (4)
C10—C11—C12—C13	−0.5 (5)	C10'—C11'—C12'—C13'	−0.4 (5)
C11—C12—C13—C14	0.2 (5)	C11'—C12'—C13'—C14'	0.9 (5)
C12—C13—C14—C9	0.1 (5)	C10'—C9'—C14'—C13'	0.3 (4)
C10—C9—C14—C13	0.0 (4)	C7'—C9'—C14'—C13'	−177.5 (3)
C7—C9—C14—C13	178.3 (3)	C12'—C13'—C14'—C9'	−0.9 (5)
C9—C7—C15—C16	−116.8 (3)	C9'—C7'—C15'—C20'	118.0 (3)
C8—C7—C15—C16	5.7 (4)	C8'—C7'—C15'—C20'	−118.3 (3)
C6—C7—C15—C16	117.6 (3)	C6'—C7'—C15'—C20'	−8.3 (3)
C9—C7—C15—C20	62.9 (3)	C9'—C7'—C15'—C16'	−61.1 (3)
C8—C7—C15—C20	−174.7 (2)	C8'—C7'—C15'—C16'	62.6 (3)
C6—C7—C15—C20	−62.7 (3)	C6'—C7'—C15'—C16'	172.6 (2)
C20—C15—C16—C17	−0.3 (4)	C20'—C15'—C16'—C17'	0.8 (4)
C7—C15—C16—C17	179.3 (2)	C7'—C15'—C16'—C17'	179.9 (3)
C15—C16—C17—C18	−0.2 (4)	C15'—C16'—C17'—C18'	−0.3 (5)
C16—C17—C18—C19	0.4 (4)	C16'—C17'—C18'—C19'	0.2 (5)
C17—C18—C19—C20	−0.1 (5)	C17'—C18'—C19'—C20'	−0.6 (5)
C18—C19—C20—C15	−0.4 (5)	C16'—C15'—C20'—C19'	−1.1 (4)
C16—C15—C20—C19	0.6 (4)	C7'—C15'—C20'—C19'	179.8 (2)
C7—C15—C20—C19	−179.0 (3)	C18'—C19'—C20'—C15'	1.0 (4)

### Hydrogen-bond geometry (Å, º)

Cg1 and Cg2 are the centroids of the C9–C14 and C15'–C20' rings, respectively.

**Table:**

*D*—H···*A*	*D*—H	H···*A*	*D*···*A*	*D*—H···*A*
C2—H2···*Cg*1^i^	1.00	2.53	3.519 (3)	171
C2′—H2′···*Cg*2^ii^	1.00	2.54	3.533 (3)	171

Symmetry codes: (i) −*x*+1, −*y*+1, −*z*+1; (ii) −*x*+2, −*y*+2, −*z*.
